# Immune infiltration is associated with distinct transcriptional states and metabolic profiles in chordomas

**DOI:** 10.1016/j.isci.2026.115668

**Published:** 2026-04-09

**Authors:** Siddh van Oost, Rick Ursem, Zeynep B. Erdem, Sara Cardoso, Sanne Venneker, Ruud van der Breggen, Inge H. Briaire-de Bruijn, Alwine B. Kruisselbrink, Wilco C. Peul, Karoly Szuhai, Robert J.P. van der Wal, Rene van Zeijl, Bram Heijs, Noel F.C.C. de Miranda, Judith V.M.G. Bovee

**Affiliations:** 1 Department of Pathology, Leiden University Medical Center, Leiden, the Netherlands; 2 Leiden Center for Computational Oncology, Leiden University Medical Center, Leiden, the Netherlands; 3 Center of Proteomics & Metabolomics, Leiden University Medical Center, Leiden, the Netherlands; 4 University Neurosurgical Center Holland, Leiden University Medical Center, Leiden, the Netherlands; 5 Department of Cell & Chemical Biology, Leiden University Medical Center, Leiden, the Netherlands; 6 Department of Orthopedic Surgery, Leiden University Medical Center, Leiden, the Netherlands; 7 Department of Pathology, Netherlands Cancer Institute, Amsterdam, the Netherlands

**Keywords:** health sciences, medicine, immunology

## Abstract

Chordomas are ultra-rare malignancies of the axial skeleton with limited treatment options. Some tumors exhibit immunogenic features and respond to checkpoint blockade, but the biological determinants remain unclear. To investigate chordoma immunogenicity, we integrated spatial transcriptomic and metabolomic profiling. GeoMx digital spatial profiling was performed on 15 chordomas, analyzing tumor and stromal compartments and comparing inflamed versus non-inflamed tumors. Consecutive sections underwent MALDI mass spectrometry imaging. Key findings were validated by immunohistochemistry. To link transcriptional states with interferon-γ, chordoma cell lines were treated with interferon-γ. Spatial profiling revealed transcriptomic differences between inflamed and non-inflamed tumors across both compartments, and immune infiltration correlated with a broader gene expression repertoire in cancer cells. MSI identified distinct metabolic signatures, including glycogen accumulation in inflamed tumors. However, interferon-γ treatment did not induce *TBXT* (brachyury) expression in chordoma cells. Together, these data reveal strong associations between transcriptional states, metabolic profiles, and immune infiltration in chordomas.

## Highlights

• Inflamed chordomas exhibit broader transcriptional repertoires & pathway activity
• Inflamed chordomas display lipid & metabolic features suggestive of oxidative stress
• Inflamed chordomas more often present high glycogen accumulation in tumor cells
• Higher expression of *TBXT* in inflamed chordomas is likely not driven by IFN-γ

## Graphical abstract

**Figure:**
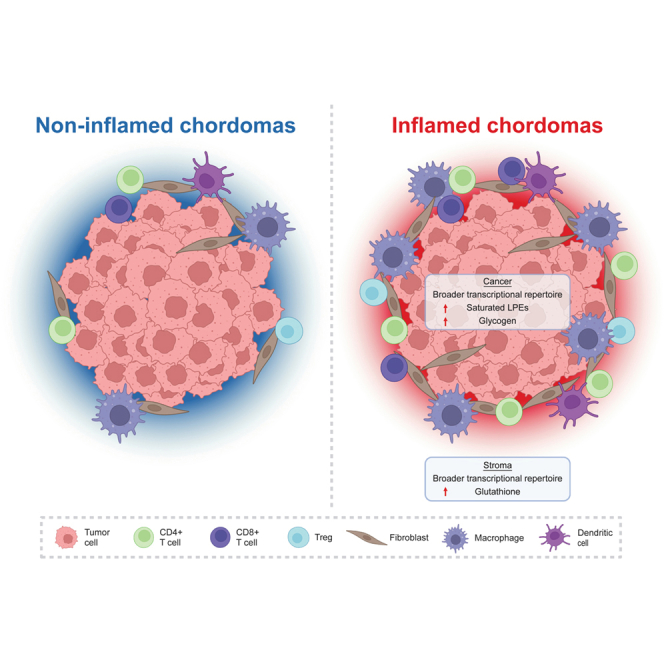

## Introduction

Chordomas are ultra-rare, malignant bone tumors demonstrating notochordal differentiation, arising almost exclusively in the axial skeleton, including the skull base, mobile spine and sacrum.^1^ The mainstay of treatment involves surgical resection and/or (proton beam) radiation.^2^^,^^3^ While (proton beam) radiation can improve local control, *en bloc* resection remains the only curative option for localized disease. Clinical management is complicated by the proximity of tumors to critical neurovascular structures, often resulting in incomplete surgical margins, high rates of local recurrence, and high morbidity.^2^^,^^3^ The lack of effective systemic therapies for advanced disease further emphasizes the urgent need for innovative and combinatory treatment strategies.^4^

Intriguingly, T cell checkpoint blockade therapy has demonstrated promising results in a subset of patients, which suggests that chordomas harbor immunogenic features.^5^^,^^6^^,^^7^ However, biomarkers predicting responsiveness to T cell checkpoint blockade remain elusive. Previously, we shed light on natural immunity in chordomas through the identification of distinct immune contextures.^8^ Together with other studies,^9^^,^^10^^,^^11^^,^^12^^,^^13^ these findings suggest a dichotomy in chordoma inflammatory profiles, with some tumors showing pronounced immune infiltrate (inflamed), while others show little or no immune activity (non-inflamed). The mechanisms underlying this variability in immunogenic features remain unclear.

To investigate correlates of immunogenicity in chordomas, we applied an integrated spatial multimodal approach combining spatial transcriptomics with spatial metabolomics. Inflamed tumors showed higher expression of immune-related and chordoma-associated genes together with broader pathway engagement in both tumor and stromal compartments, consistent with coordinated biological states between tumor cells and the immune microenvironment. Furthermore, inflamed chordomas displayed distinct metabolic profiles. Collectively, these findings highlight distinct tumor cell states associated with immune infiltration in chordomas and could potentially inform patient stratification, particularly in the context of immunotherapy.

## Results

### Inflammation is associated with distinct transcriptional states

**Figure 1:**
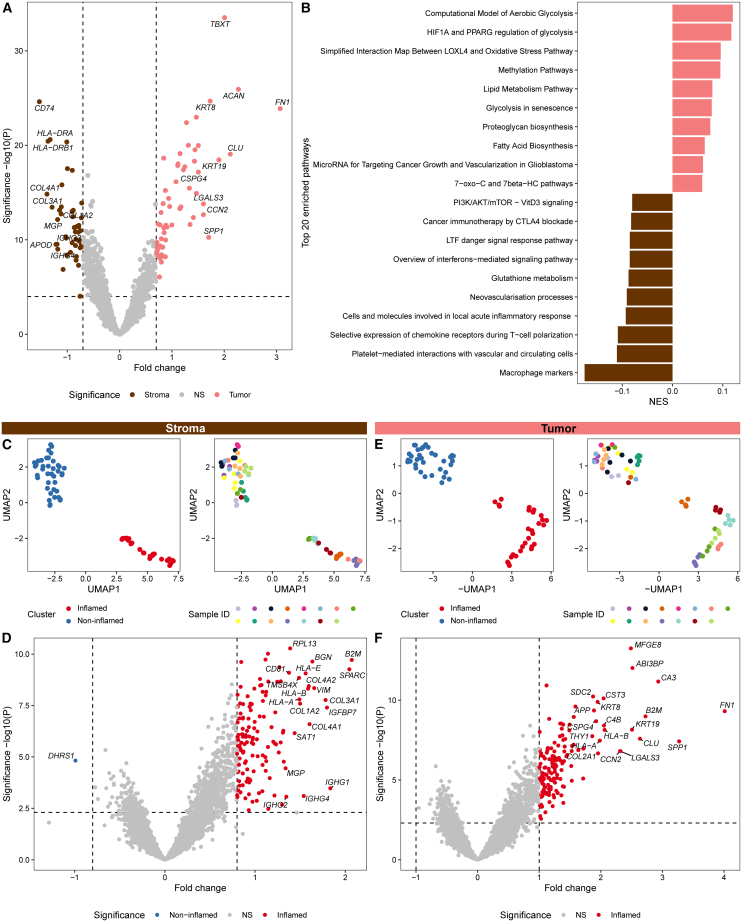
Inflammation is associated with distinct transcriptional states (A) Volcano plot presenting the differential gene expression between stroma (*n* = 63) and tumor (*n* = 63) segments. The top ten DEGs per segment, ranked by fold change, are highlighted. Statistical significance was determined using a linear mixed model (LMM) correcting for sample ID. See also Figure S2. (B) Bar plot of the normalized enrichment score (NES) for the top ten differentially enriched pathways per compartment, based on a curated WikiPathways list. Statistical significance was assessed using an LMM correcting for sample ID. (C) UMAP visualizations of stroma segments using the top 20% most variably expressed genes. Clusters were defined via graph-based clustering. Segments are colored by cluster (left) and sample ID (right). (D) Volcano plots comparing non-inflamed (*n* = 40) versus inflamed (*n* = 23) stroma segments. The top 20 DEGs are highlighted per cluster. Statistical significance was determined using an LMM correcting for sample ID. See also Figure S3. (E) UMAP visualizations of tumor segments using the top 20% most variably expressed genes. Clusters were defined via graph-based clustering. Segments are colored by cluster (left) and sample ID (right). (F) Volcano plots comparing non-inflamed (*n* = 34) versus inflamed (*n* = 29) tumor segments. The top 20 DEGs are highlighted per cluster. Statistical significance was determined using an LMM correcting for sample ID. See also Figure S5.

To investigate transcriptional profiles of both tumor and stromal compartments that associate with immune infiltration in chordomas, GeoMx was performed on frozen sections from 15 conventional chordomas. For each sample, two to five regions of interest (ROIs) along the tumor-stroma interface were selected (Figure S1). Gene expression profiles were then delineated into tumor and stroma compartments based on pan-cytokeratin expression. Differential gene expression analysis was performed to compare tumor segments with stroma segments (Figure 1A; Figure S2). This analysis confirmed the enrichment of chordoma-associated genes in tumor segments, including *TBXT*, *KRT8*, and *KRT19*, and immune- and stroma-related genes in stroma segments, such as genes encoding collagens, human leukocyte antigen (HLA) class II molecules, or immunoglobulins (Figure 1A; Figure S2). In line with the established transcriptional role of *TBXT*, several tumor-enriched genes correspond to known *TBXT* targets,^14^ including *ACAN* and *CCN2* (Figure 1A), with additional targets shown in Figure S2. Next, biological pathways were explored through a single-sample gene set enrichment analysis (ssGSEA) for all segments using an in-house curated WikiPathways list. Accordingly, differential pathway analysis comparing tumor with stroma segments confirmed that tumor segments were enriched for cancer-associated pathways, such as glycolysis, while stroma segments showed enrichment of immune- and stroma-related pathways like inflammatory signaling and neovascularization (Figure 1B).

Following this general characterization, tumor and stroma segments were interrogated separately to investigate the association between cancer cell-specific gene expression and immune infiltration. Using the top 20% most variably expressed genes (1,680 genes), graph-based clustering identified two distinct groups of stroma segments, which were visualized with uniform manifold approximation and projection (UMAP) (Figure 1C). Differential gene expression analysis indicated that these clusters reflected inflamed and non-inflamed stroma segments due to the enrichment of immune-related genes or the lack thereof (Figure 1D; Figure S3). These genes indicated enrichment of (plasma) B cells (*IGHG1*, *IGHG4*), antigen-presenting cells (*HLA-DRA*, *HLA-DQB1*), macrophages (*CD68*, *LYZ*), fibroblasts (*COL1A2*, *COL3A1*), endothelial cells (*ACTA2*, *COL4A1*), complement (*C1R*, *C4B*), and T cells (*TRAC*). This pattern was accompanied by increased numbers of captured nuclei and detected genes in inflamed segments (Figures S4A and S4B), reflecting increased immune and stromal cell presence together with a broader detected gene repertoire.

Building on this clear dichotomy within the stroma segments, tumor segments were also clustered into two groups using graph-based clustering of the top 20% most variably expressed genes and visualized with UMAP (Figure 1E). Interestingly, these tumor clusters closely mirrored the inflammation-associated pattern observed in the stroma clusters, with 14 of the 15 samples grouping concordantly across both compartments. Because multiple ROIs were analyzed per sample, intratumoral heterogeneity was observed and both inflamed and non-inflamed segments could be present within a single tumor. For downstream analyses requiring a sample-level classification, tumors were therefore assigned an overall classification based on their predominant profile, defined as the cluster identity represented by the majority of segments within that sample. In rare cases, stromal and tumor compartments from the same sample displayed diverging profiles. This was observed for sample L6951 (light green), in which stromal segments clustered with the non-inflamed group whereas tumor segments clustered with the inflamed group. Histological evaluation revealed extensive radiotherapy-associated stromal fibrosis with limited immune infiltration, while viable tumor areas remained present. Supporting these observations, previously generated imaging mass cytometry data from the same tumors showed increased T cell infiltration in the inflamed tumor cluster compared with the non-inflamed cluster (Figure S4C). As expected, genes associated with immunity (*B2M*, *C1S*, *C4B*, *HLA-A*, *HLA-B*, *HLA-E*, and *IFITM3*) showed higher expression in the inflamed tumor segments than in the non-inflamed segments (Figure 1F; Figure S5). More intriguingly, several chordoma-associated genes (*TBXT*, *CA3*, *CCN2*, *CD24*, *COL2A1*, *KRT19*, and *SOX9*) also exhibited higher expression in the inflamed tumor segments than in the non-inflamed segments (Figure 1F; Figure S5). Unlike the stromal compartment, the number of captured nuclei did not differ between inflamed and non-inflamed tumor segments (Figure S4D), whereas the number of detected genes was higher in the inflamed segments (Figure S4E). Together with the overall enrichment of differentially expressed genes (DEGs) in the inflamed segments, these results indicate that chordoma cells in inflamed segments exhibit a broader gene expression repertoire than those in non-inflamed segments, consistent with a distinct tumor cell state associated with immune infiltration.

To interrogate which biological pathways were associated with the observed inflamed phenotype, a differential ssGSEA was performed comparing inflamed with non-inflamed segments for both tumor and stromal compartments. To facilitate biological interpretation, the top 10% of differentially enriched pathways (22 pathways) based on adjusted *p* values are shown for the stroma and tumor compartments (Figures S6A and S6B). Within the stroma compartment, inflamed segments exhibited enrichment of pathways related to immune activation and regulation, together with pathways involved in extracellular matrix organization, angiogenesis, and cellular stress responses (Figure S6A). In contrast, non-inflamed stroma segments showed few differentially enriched pathways. A similar pattern was observed within the tumor segments (Figure S6B). Inflamed tumor segments were enriched for a broad range of biological processes, including cellular metabolism, RNA processing and protein turnover, extracellular matrix production, and immune-related signaling. In contrast, non-inflamed tumor segments showed enrichment of only a limited number of pathways. Together, these pathway-level differences mirror the gene-level patterns observed between inflamed and non-inflamed segments, indicating broader pathway activity in inflamed segments across both tumor and stromal compartments.

### Inflammation is associated with distinct lipid and metabolic profiles

**Figure 2:**
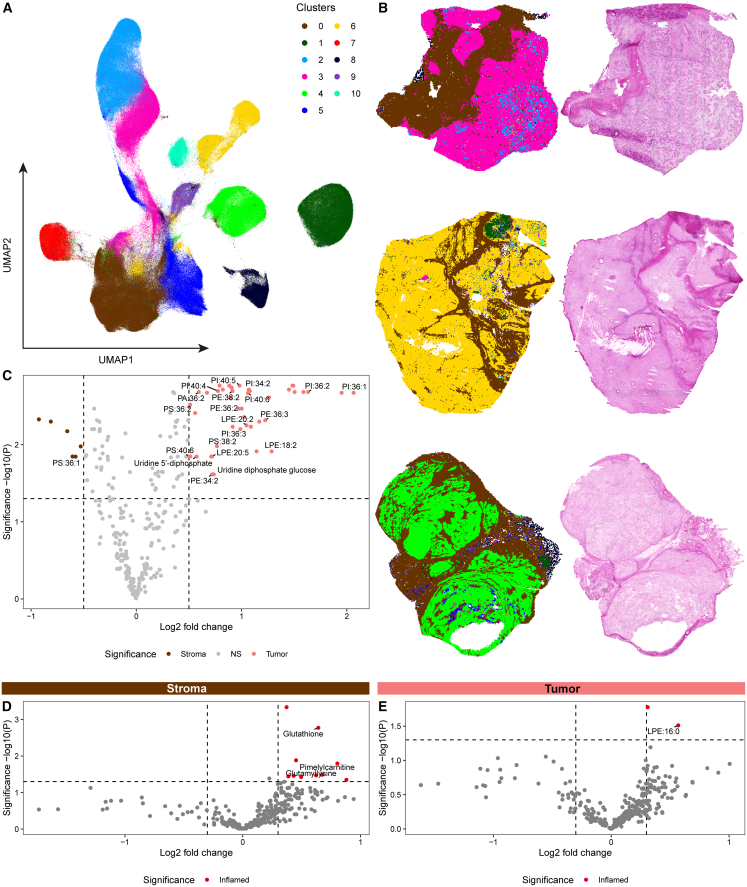
Spatial lipid and metabolite profiles in relation to inflammation (A) UMAP visualization of all measured lipid profiles (*n* = 1,223,810 pixels) from the MALDI-MSI data. Clusters were identified through Louvain clustering. (B) Spatial visualization of the identified clusters (stroma = brown; tumor = pink, yellow, and green) next to corresponding histopathological images for three samples. (C) Volcano plot presenting the differential lipid/metabolite abundance analysis comparing tumor (*n* = 13) with stroma (*n* = 13) regions. Significance was assessed by paired Student’s *t* tests, which were corrected for multiple testing with the Benjamini-Hochberg method. (D and E) Volcano plots presenting the differential lipid/metabolite abundance analysis comparing non-inflamed (*n* = 7) with inflamed (*n* = 6) samples, for tumor regions (D) and for stromal regions (E). Significance was determined by unpaired Student’s *t* tests without multiple testing correction.

In parallel with the spatial transcriptomic analyses, we explored the spatial metabolic and lipid profiles of chordomas in relation to immune infiltration using MALDI mass spectrometry imaging (MSI) at 20 μm resolution on whole slides from consecutive frozen sections of the same samples analyzed by GeoMx. This analysis included sequential tissue sections from 13 of the 15 patients; for two patients, three recurrent tumor samples were additionally included to assess whether lipid profiles were preserved over the course of disease. For the data-driven annotation of the tissues, all measured lipid profiles were visualized using UMAP (Figure 2A) and, following clustering, were mapped back to their original spatial coordinates. By aligning with histopathological images, the lipid clusters enabled differentiation between tumor and stromal regions (Figure 2B). This facilitated comparative analyses both between tumor and stroma as well as between inflamed and non-inflamed regions within each compartment. Following classification of the lipid clusters as either tumor- (clusters 1–10) or stroma-associated (cluster 0), tumor regions exhibited strong patient-specific profiles, while stromal profiles were relatively similar across patients (Figures 2A and 2B). Notably, in patients for whom multiple tumor samples were available, tumor profiles clustered closely with those of previous tumors from the same patient, suggesting that these patient-specific lipid signatures appear largely preserved over time. Differential analysis showed that tumor areas were enriched for a diverse set of lipids, predominantly phosphatidylinositol (PI) species, along with some lysophosphatidylethanolamines (LPE), phosphatidic acid (PA), phosphatidylethanolamine (PE), and phosphatidylserine (PS) species, while stromal regions were enriched for only a single PS species (Figure 2C). Notably, tumors were enriched in polyunsaturated lipids (with more than two double bonds), suggesting more flexible and dynamic cell membranes. In contrast, the PS species enriched in stroma (PS[36:1]) is more saturated, containing only a single double bond, consistent with more rigid and stable membranes.

Next, we compared inflamed and non-inflamed chordomas, as defined earlier by data-driven clustering of GeoMx segments, using differential abundance analysis within each compartment (Figures 2D and 2E). The resulting profiles mirrored the GeoMx findings, although differences were less pronounced: inflamed chordomas displayed a greater number of differentially abundant lipids and metabolites in both tumor and stromal regions than non-inflamed chordomas. In particular, glutathione was enriched in the inflamed stroma, while the saturated lipid species LPE (16:0) was elevated in inflamed tumor regions. Together, these findings indicate that inflamed chordomas are associated with distinct lipid and metabolic profiles compared with non-inflamed chordomas.

### Inflammation is associated with increased glycogen accumulation and proliferation

**Figure 3:**
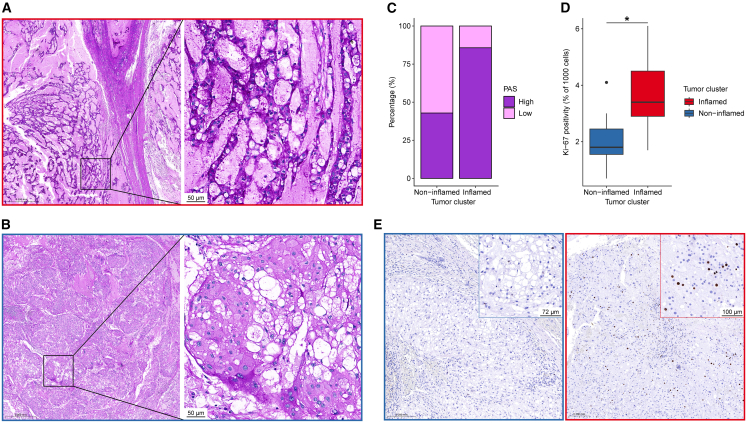
Glycogen accumulation and proliferation are increased in inflamed chordomas (A and B) Representative PAS staining images of an inflamed chordoma (A), classified as high PAS staining, and a non-inflamed chordoma (B), classified as low PAS staining. Scale bars represent 0.5 mm in the left images and 50 μm in the magnified images on the right. (C) Bar plots displaying the percentage of chordomas with high and low PAS staining, grouped by tumor cluster (non-inflamed: *n* = 7; inflamed: *n* = 7). (D) Boxplots presenting the percentage of Ki-67^+^ tumor cells in proliferative hotspot areas, grouped by tumor cluster (non-inflamed: *n* = 7; inflamed: *n* = 7). Statistical significance was assessed using an unpaired Student’s *t* test: ∗*p* < 0.05. (E) Representative Ki-67 immunohistochemistry showing Ki-67 expression in a non-inflamed chordoma (left) and an inflamed chordoma (right). Scale bars represent 0.2 mm in the main images, 72 μm in the magnified image on the left, and 100 μm in the magnified image on the right.

The MSI analysis comparing tumor with stroma revealed that the metabolite uridine diphosphate (UDP)-glucose, the key substrate for glycogen synthase, was enriched in tumor regions compared to stroma (Figure 2C). Consistently, the GeoMx analysis showed increased expression of two glycogen synthesis–related genes, *PPP1R3C* and *UGP2*, in tumor segments from inflamed samples (as defined earlier by data-driven clustering of GeoMx segments; Figure S5). *PPP1R3C* encodes a subunit of the protein phosphatase 1 (PP1) complex, which activates glycogen synthase while inhibiting glycogen phosphorylase, thereby promoting glycogen storage. *UGP2* encodes UDP-Glucose Pyrophosphorylase 2, an enzyme that catalyzes the conversion of glucose-1-phosphate and uridine triphosphate (UTP) into UDP-glucose. Supporting these findings, Periodic acid-Schiff (PAS) and diastase-PAS (dPAS) staining demonstrated abundant glycogen accumulation in tumor cells of most chordomas, with inflamed tumors generally exhibiting stronger staining intensity and greater glycogen abundance than non-inflamed tumors (Figures 3A–3C). Collectively, these results indicate that glycogen synthesis pathways are active in chordomas, and that inflamed tumors exhibit higher glycogen accumulation than non-inflamed tumors.

Following this, we examined whether the distinct tumor cell state observed in inflamed chordomas, together with increased glycogen accumulation, was associated with differences in tumor cell proliferation. Although proliferation-associated genes were not detected in the GeoMx dataset, likely due to their low expression and the limitations of NanoString’s Whole Transcriptome Atlas (WTA), immunohistochemistry for Ki-67 showed that inflamed tumors were more proliferative than the non-inflamed tumors (Figures 3D and 3E). Nonetheless, proliferation remained very low compared with that observed in carcinomas or high-grade sarcomas, with only two samples showing Ki-67 positivity above 5%.

### Interferon-γ does not induce *TBXT* expression in chordoma cell lines

**Figure 4:**
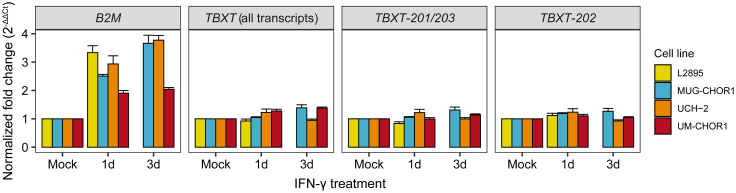
*TBXT* expression is not induced by IFN-γ Normalized expression fold change for *B2M* and the *TBXT* isoforms across chordoma cell lines (*n* = 4) and treatment conditions. Average Ct values from technical triplicates were normalized to the housekeeping genes *HPRT1* and *TBP*. Data are represented as mean ± SD. Abbreviations: 1day = 1 day; 3days = 3 days. See also Figure S7.

To determine whether the broader transcriptional repertoire observed in inflamed chordomas—including increased expression of *TBXT* and its target genes—could be driven by interferon-γ (IFN-γ) signaling, as suggested by the enrichment of IFN-γ-responsive genes (e.g., HLA class I) in inflamed tumor regions and activation of type II interferon signaling in the stroma, we investigated whether IFN-γ modulates expression of *TBXT*, the master regulator of chordoma biology. Multiple chordoma cell lines were treated with IFN-γ, and expression of *B2M* (positive control for IFN-γ signaling) and *TBXT* was assessed by quantitative polymerase chain reaction (qPCR), with isoform-specific primers for *TBXT* (Table 1). Ct values were normalized to two established chordoma housekeeping genes, *HPRT1* and *TBP*,^15^^,^^16^ which showed stable expression across all treatment conditions (Figure S7). As expected, IFN-γ treatment robustly increased *B2M* expression in all cell lines (Figure 4). In contrast, expression of *TBXT* isoforms remained unchanged across cell lines, indicating that *TBXT* expression is unlikely to be regulated by IFN-γ signaling.

## Discussion

In this study, we applied an integrated spatial approach to explore correlates of immunogenicity in chordoma. Our findings revealed a clear dichotomy between inflamed and non-inflamed chordomas across both tumor and stromal compartments. Inflamed tumors exhibited higher expression of immune-related genes, such as those encoding HLA class I molecules and complement factors, as well as chordoma-associated genes, including *TBXT*, *SOX9*, and *COL2A1*, within the tumor cell compartment. These transcriptional differences were accompanied by distinct metabolic profiles and increased glycogen accumulation, indicating a distinct tumor cell state associated with, and potentially interacting with, immune infiltration. To examine whether inflammatory signaling could contribute to the broader transcriptional repertoire observed in inflamed tumors, we investigated whether IFN-γ modulates expression of *TBXT*, the master regulator of chordoma biology. While chordoma cells were responsive to IFN-γ, *TBXT* expression remained unchanged following IFN-γ treatment, suggesting that IFN-γ signaling is unlikely to account for the higher *TBXT* expression observed in inflamed tumors. Nevertheless, other cytokines or mediators released by infiltrating immune or stromal cells may still contribute to the tumor cell states observed in inflamed chordomas.

These findings extend prior work, including our own, that identified distinct immune contextures in chordoma,^8^^,^^9^^,^^10^^,^^11^^,^^12^^,^^13^ and highlight a strong association between tumor cell gene expression patterns and immune infiltration. Although the causal link remains unresolved, differences in *TBXT* expression may contribute to the heterogeneity observed in tumor phenotypes and immune contextures. Such variability could be shaped by underlying genetic or epigenetic factors. For instance, duplications or amplifications of the *TBXT* locus have been reported in 15%–30% of chordoma patients.^17^^,^^18^^,^^19^ In addition, chordomas are transcriptionally addicted to super-enhancer-driven *TBXT* expression,^20^^,^^21^^,^^22^ and variation in enhancer strength or chromatin state between tumors may contribute to differences in *TBXT* expression levels. Because *TBXT* regulates a broad range of transcriptional programs,^14^^,^^23^ its differential activity may contribute to the broader transcriptional repertoire observed in inflamed chordomas. Of note, *TBXT* has also been linked to interferon-α response networks in chordomas,^23^ although this observation derives from a single study and its future implications for chordoma immunity remain to be established.

In addition to its role as a master regulator, *TBXT* has been identified as an immunogenic antigen in chordoma. Brachyury-derived peptides have been shown to expand T cells *in vitro*,^24^^,^^25^ and vaccination strategies targeting brachyury have elicited *TBXT*-specific T cell responses in a subset of chordoma patients.^26^^,^^27^ Furthermore, a recent conference abstract presented at the SITC 39^th^ Annual Meeting (2024) reported naturally presented HLA peptides derived from brachyury across common alleles, covering 80% of their patient population (Pan K., Chiu Y., Patel H.A., Huang E., MacCoss M., Yee C. 387 Identification of novel T cell receptor [TCR] target to brachyury for the development of T cell-based immunotherapy of chordoma and other solid tumors. *Journal for ImmunoTherapy of Cancer*. 2024;12:. https://doi.org/10.1136/jitc-2024-SITC2024.0387). Together, these studies highlight the immunogenic potential of *TBXT* as an antigen and support further investigation into how *TBXT*-directed immune responses can be effectively leveraged in chordoma.

Our spatial metabolomics analysis revealed that tumor regions of chordoma were enriched in polyunsaturated lipids. Because these lipids contain multiple double bonds, they are particularly susceptible to lipid peroxidation—a process in which reactive oxygen species attack fatty acids, generating toxic radicals that compromise membrane integrity and can trigger ferroptosis, a regulated form of cell death.^28^ Consistent with this vulnerability, the Chordoma Foundation recently reported preclinical evidence suggesting ferroptosis sensitivity in chordoma cell lines (Chordoma Foundation. [2024]. Ferroptosis in chordoma. Figshare. https://doi.org/10.6084/m9.figshare.26517532), supporting the relevance of lipid peroxidation pathways in this disease. In parallel, inflamed tumors were enriched for a saturated LPE species (16:0) in tumor regions and glutathione in stromal regions. Glutathione, a key antioxidant, and saturated lipids, which are less prone to peroxidation, collectively point toward adaptive responses to oxidative stress. Together, these findings suggest that inflamed tumors may experience increased oxidative stress, potentially reflecting heightened tumor cell activity, immune infiltration, or both.^29^ Further studies will be required to clarify how oxidative stress relates to tumor cell states and immune infiltration in chordoma.

In addition to lipid and antioxidant adaptations, inflamed chordomas also exhibited increased glycogen accumulation, accompanied by higher expression of glycogen synthesis-related genes. Glycogen serves as a critical energy reserve that supports cell survival under hypoxic conditions,^30^ and its increased accumulation in inflamed tumors may therefore provide a metabolic advantage that sustains tumor cell activity and viability within an immune-infiltrated microenvironment. Glycogen storage is, however, a recognized feature of chordomas,^31^ likely associated with their notochordal differentiation.^32^ This notion is supported by the observation that *PPP1R3C* is a transcriptional target of *TBXT*.^14^ Accordingly, the increased glycogen accumulation observed in inflamed chordomas may reflect enhanced *TBXT*-associated transcriptional programs rather than representing solely a microenvironmental-driven metabolic adaptation.

Going forward, it will be important to identify proxies or biomarkers that reflect immunogenic potential, enabling broader clinical translation. Notably, recent work by Crombé et al. demonstrated that high ^18^F-fluorodeoxyglucose (FDG) uptake correlates with increased immune cell densities in high-grade soft tissue sarcomas.^33^ Consistent with our findings, this suggests that metabolic imaging approaches such as FDG positron emission tomography could potentially serve as non-invasive tools to identify immunogenic chordoma subsets and guide patient stratification for immunotherapeutic strategies. This concept, however, will require validation in larger, ideally multi-institutional, cohorts to confirm its clinical utility.

In conclusion, our integrated spatial approach demonstrates that inflamed chordomas exhibit distinct transcriptional and metabolic features, marked by a broader transcriptional repertoire, adaptations to oxidative stress, and increased glycogen accumulation. These findings highlight a close association between tumor cell states, metabolic adaptations, and immune infiltration. While the causal relationships among these processes remain to be established, they point to metabolic and transcriptional states as potential indicators of tumor immunogenicity. Ultimately, such integrated molecular and spatial insights may guide the development of biomarkers and inform patient stratification for future immunotherapeutic strategies.

### Limitations of the study

This study has several limitations. First, the cohort predominantly comprises sacrococcygeal chordomas. This reflects the clinical reality that sacrococcygeal tumors are generally larger and more frequently resected *en bloc* than clival or spinal chordomas, resulting in greater amounts of frozen material suitable for spatial multi-omics analyses. Nevertheless, prior work,^34^ including our own,^8^ suggests that the immune microenvironment does not substantially differ across anatomical locations, which is supported here by the comparable distribution of clival and spinal samples across inflammatory clusters. Finally, functional experiments were limited to IFN-γ stimulation of chordoma cell lines. Although IFN-γ robustly induced *B2M*, *TBXT* expression remained stable across cell lines, indicating that *TBXT* is not regulated by IFN-γ under these conditions. This analysis was, however, restricted to a limited set of genes and a single cytokine. Expanding future studies to include broader panels of inflammatory mediators and downstream transcriptional programs will be important to determine whether the distinct tumor cell state observed in inflamed chordomas is driven by alternative microenvironmental cues or reflects intrinsic tumor biology.

## Resource availability

### Lead contact

• Requests for further information and resources should be directed to and will be fulfilled by the lead contact, Judith V.M.G. Bovee (j.v.m.g.bovee@lumc.nl).

### Materials availability

• There are restrictions to the availability of the primary chordoma cell line L2895 because institutional and biobank approval for distribution outside our institute is currently pending.

### Data and code availability

• The utilized imaging mass cytometry data are available as raw data in BioStudies at S-BIAD830 or as processed data on our lab’s GitHub (https://github.com/deMirandaLab/multimodal-profiling-of-chordomas). The raw and processed GeoMx digital spatial profiling data and the processed MALDI-MSI data generated in this study are publicly available on Figshare (https://figshare.com/s/4d52d0129b9e0f9c7a8c). The raw MALDI-MSI data are publicly available in MassIVE at MSV000100742.
• The R code used to process the data from this study is also published on our lab’s GitHub (https://github.com/deMirandaLab/spatial-profiling-of-chordomas).
• All other data are available from the corresponding author upon reasonable request.

## Acknowledgments

We acknowledge the Utrecht Sequencing Facility (USEQ) for providing sequencing service and data. USEQ is subsidized by the University Medical Center Utrecht and The Netherlands X-omics Initiative (NWO project 184.034.019). J.V.M.G.B. is the recipient of a 10.13039/100000884Cancer Research Institute/Chordoma Foundation CLIP Grant (CRI5106). This work was further funded by the intramural Leiden Center for Computational Oncology strategic fund. N.F.C.C.d.M. is funded by the European Research Council (10.13039/100010663ERC) under the European Union’s Horizon 2020 Research and Innovation Program (grant agreement no. 852832) and by the VIDI ZonMW (project number: 09150172110092). The graphical abstract was created in https://BioRender.com.

## Author contributions

Conceptualization, S.v.O., N.F.C.C.d.M., and J.V.M.G.B.; data curation, S.v.O. and R.U.; software, S.v.O., R.U., and B.H.; formal analysis, S.v.O., R.U., R.v.d.B., and B.H.; validation, S.v.O., R.U., Z.B.E., S.C., S.V., R.v.d.B., and A.B.K.; visualization, S.v.O. and R.U.; methodology, S.v.O., R.U., S.C., S.V., R.v.d.B., I.H.B.-d.B., A.B.K., B.H., N.F.C.C.d.M., and J.V.M.G.B.; investigation, S.v.O., R.U., Z.B.E., S.V., R.v.d.B., I.H.B.-d.B., A.B.K., R.v.Z., B.H., N.F.C.C.d.M., and J.V.M.G.B.; writing – original draft, S.v.O., R.U., S.V., A.B.K., N.F.C.C.d.M., and J.V.M.G.B.; writing – review and editing, all authors; project administration, S.v.O.; funding acquisition, S.v.O., N.F.C.C.d.M., and J.V.M.G.B.; resources, W.C.P., K.S., and R.J.P.v.d.W.; supervision, N.F.C.C.d.M. and J.V.M.G.B.

## Declaration of interests

The authors declare no competing interests.

## Declaration of generative AI and AI-assisted technologies in the writing process

During the preparation of this work the authors used ChatGPT in order to improve the readability and language of the manuscript. After using this tool/service, the authors reviewed and edited the content as needed and take full responsibility for the content of the published article.

## STAR★Methods

### Key resources table

**Table:** 

REAGENT or RESOURCE	SOURCE	IDENTIFIER
**Antibodies**		

pan-cytokeratin (AE-1/AE-3)	Novus Biologicals	Cat#NBP2-33200AF532; RRID:AB_2924722
CD45 (2B11 + PD7/26)	Novus Biologicals	Cat#NBP2-34528AF594; RRID:AB_3287665
Ki-67 (MIB-1, FLEX RTU)	Dako	Cat#GA626; RRID:AB_2687921

**Biological samples**		

Human chordoma tumor tissue	Leiden Unviersity Medical Center	N/A

**Chemicals, peptides, and recombinant proteins**		

Syto13	Thermo Fisher Scientific	Cat#S7575
*N*-(1-naphthyl) ethylenediamine dihydrochloride (NEDC)	Sigma-Aldrich	https://www.sigmaaldrich.com/NL/en/product/aldrich/n9125?srsltid=AfmBOoo90g2yMEhQywjHzlYcGWeExUv2v492QealgsKEo9Q0BX_IDVcc; CAS Number: 1465-25-4
MeOH (Methanol)	Actu-All Chemicals	Cat#1132122500; CAS Number: 67-56-1
Acetonitrile	Actu-All Chemicals	Cat#1012192500; CAS Number: 75-05-8
IMDM (Medium)	Gibco	https://www.thermofisher.com/order/catalog/product/12440053
RPMI 1640 (Medium)	Gibco	https://www.thermofisher.com/order/catalog/product/11875093
FBS (Fetal-Bovine Serum)	Sigma-Aldrich	Cat#F7524
Insulin-Transferrin-Selenium (ITS-G)	Gibco	Cat#41400045
Collagen Type I	Ibidi	Cat#50202
IFN-γ (interferon-gamma)	Immunotools	Cat#11343536

**Critical commercial assays**		

Human NGS Whole Transcriptome (WTA) RNA probe set (v1.0)	NanoString Technologies	https://nanostring.com/wp-content/uploads/PB_MK3683_GeoMx-WTA_r9.pdf
Artisan PAS Stain Kit	Artisan, Agilent	Cat#AR165
Artisan Alpha-Amylase reagent	Artisan, Agilent	Cat#AR171
NucleoSpin RNA Mini kit	Macherey-Nagel	https://www.mn-net.com/nucleospin-rna-mini-kit-for-rna-purification-740955.50
Transcriptor First Strand cDNA Synthesis Kit	Roche	https://lifescience.roche.com/global/en/products/others/transcriptor-first-strand-cdna-synthesis-kit-3683912.html
iQ SYBR Green Supermix	Bio-Rad	https://www.bio-rad.com/en-nl/product/iq-sybr-green-supermix?ID=M8G3SJ15

**Deposited data**		

GeoMx DSP	Leiden University Medical Center; This paper	Raw and processed: https://figshare.com/s/4d52d0129b9e0f9c7a8c
MALDI-MSI	Leiden University Medical Center;This paper	Raw: MassIVE: MSV000100742; Processed: https://figshare.com/s/4d52d0129b9e0f9c7a8c
Imaging Mass Cytometry	Leiden University Medical Center	Raw: BioStudies: S-BIAD830; Processed: https://github.com/deMirandaLab/multimodal-profiling-of-chordomas

**Experimental models: Cell lines**		

L2895 (in-house; human primary chordoma; female; age65)	Leiden University Medical Center; This paper	N/A
MUG-CHOR1 (recurrent chordoma; female; age57)	Chordoma Foundation	https://www.chordomafoundation.org/researchers/disease-models/mug-chor1/; RRID:CVCL_9277
UCH2 (recurrent chordoma; female, age72)	Chordoma Foundation	https://www.chordomafoundation.org/researchers/disease-models/u-ch2/; RRID:CVCL_4989
UM-CHOR1 (primary chordoma; male; age66)	Chordoma Foundation	https://www.chordomafoundation.org/researchers/disease-models/um-chor1/; RRID:CVCL_1D68

**Oligonucleotides**		

*B2M* primers	Integrated DNA Technologies	See Table 1
*HPRT1* primers	Integrated DNA Technologies	See Table 1
*TBP* primers	Integrated DNA Technologies	See Table 1
*TBXT-202* isoform primers	Integrated DNA Technologies	See Table 1
*TBXT-201/203* isform primers	Integrated DNA Technologies	See Table 1
*TBXT* (all transcripts) primers	Integrated DNA Technologies	See Table 1

**Software and algorithms**		

Original R code	Leiden University Medical Center; This paper	https://github.com/deMirandaLab/spatial-profiling-of-chordomas
R (v.4.4.0)	R Project for Statistical Computing	https://www.r-project.org; RRID:SCR_001905
GeomxTools (v.3.8.0)	NanoString Technologies	https://github.com/Nanostring-Biostats/GeomxTools/; RRID:SCR_023424
ggplot2 (v.3.5.1)	R Project for Statistical Computing	https://cran.r-project.org/web/packages/ggplot2/index.html; RRID:SCR_014601
ComplexHeatmap (v.2.20.0)	Bioconductor	https://bioconductor.org/packages/release/bioc/html/ComplexHeatmap.html; RRID:SCR_017270
GSVA (v.1.52.3)	Bioconductor	https://www.bioconductor.org/packages/release/bioc/html/GSVA.html; RRID:SCR_021058
WikiPathways (in-house curated)	WikiPathways	http://wikipathways.org/; RRID:SCR_002134
bluster (v.1.14.0)	Bioconductor	https://www.bioconductor.org/packages/release/bioc/html/bluster.html
SCiLS Lab (v.2023b 11.01.14623)	Bruker Daltonics	http://scils.de/software/; RRID:SCR_014426
mMass (v.5.5.0)	My Biosoftware	https://mybiosoftware.com/mmass-5-4-0-open-source-mass-spectrometry-tool.html
R (v.4.1)	R Project for Statistical Computing	https://www.r-project.org; RRID:SCR_001905
MALDIquantForeign (v.0.13)	R Project for Statistical Computing	https://cran.r-project.org/web/packages/MALDIquantForeign/index.html
MALDIquant (v.1.20)	R Project for Statistical Computing	https://cran.r-project.org/web/packages/MALDIquant/index.html
Seurat (v.4.2)	Satija Lab	https://satijalab.org/seurat/get_started.html; RRID:SCR_016341
QuPath (v.0.3.1)	QuPath	https://qupath.github.io/; RRID:SCR_01825
CFX Maestro software (v.5.3.022.1030)	Bio-Rad	Cat#12013758

**Other**		

GeoMx Digital Spatial Profiler	NanoString Technologies	https://www.nanostring.com/products/geomx-digital-spatial-profiler/geomx-dsp-overview/; RRID:SCR_021660
Indium-tin-oxide (ITO)-coated glass slides	VisionTek Systems	https://www.visionteksystems.co.uk/ito-glass.htm
HTX M3+ sprayer	HTX Technologies	https://www.htximaging.com/htx-m3-sprayer; RRID:SCR_023731
rapifleX MALDI-TOF/TOF system	Bruker Daltonics	https://www.bruker.com/en/products-and-solutions/mass-spectrometry/maldi-tof/rapiflex.html; RRID:SCR_026525
15T solariX MALDI-FTICR mass spectrometer	Bruker Daltonics	https://www.bruker.com/en/products-and-solutions/mass-spectrometry/mrms/solarix.html; RRID:SCR_027095
ZEISS Axio Scan.Z1 slide scanner	ZEISS	https://www.ctk-instruments.com/Zeiss_AxioScanZ1-brochure.pdf; RRID:SCR_020927
Artisan Link Pro Special Staining System	Dako, Agilent	https://www.agilent.com/en/product/special-stains/automated-special-staining/artisan-link-pro-special-staining-system-776828; RRID:SCR_019467
C1000 Touch Thermal Cycler	Bio-Rad	https://www.bio-rad.com/en-nl/product/c1000-touch-thermal-cycler?ID=LGTW9415; RRID:SCR_019688
CFX384 Touch Real-Time PCR Detection System	Bio-Rad	https://www.bio-rad.com/en-nl/product/cfx384-touch-real-time-pcr-detection-system?ID=LJB22YE8Z; RRID:SCR_018057
384-well Hard-Shell thin-wall PCR plates	Bio-Rad	Cat#HSP3805
Microseal 'B' adhesive film	Bio-Rad	Cat#MSB1001

### Experimental model and study participant details

#### Patient samples

Snap-frozen tumor tissue was available for 18 samples from 15 patients with conventional chordoma (2 clival, 2 spinal and 11 sacrococcygeal), all of whom were part of a previously described cohort.^8^ An overview of the samples is provided in Table S1. All samples included in this study were derived from surgical resection specimens, including three from patients who had received neoadjuvant radiotherapy. Samples were pseudo anonymized and handled according to the ethical guidelines outlined in ‘Code for Proper Secondary Use of Human Tissue in The Netherlands’ by the Dutch Federation of Medical Scientific Societies. A waiver of consent was obtained from the medical ethical evaluation committee (Medisch-Ethische Toetsingscommissie Leiden Den Haag Delft; protocol number: B17.036 and B20.067). Therefore, this study adheres to the Declaration of Helsinki.

#### Cell lines

One chordoma cell line from a primary tumor was established in-house (L2895) and additional chordoma cell lines were acquired via the Chordoma Foundation, including MUG-CHOR1, UCH2, and UM-CHOR1. All chordoma cell lines were cultured in IMDM/RPMI 1640 (4:1, Gibco) supplemented with 10% non-inactivated FBS (F7524, Sigma-Aldrich) and 1% Insulin-Transferrin-Selenium (ITS-G, Gibco).

### Method details

#### GeoMx digital spatial profiling

Slides were prepared in a cryostat and kept on dry-ice. Tissue sections of 5 μm were thaw-mounted onto pre-cooled slides to prevent RNA degradation and then stored at -80°C until use. Slide preparation was performed by the Utrecht Sequencing Facility (Utrecht, The Netherlands) according to NanoString’s protocol. In brief, slides were fixed using 10% neutral-buffered formalin, followed by overnight *in situ* hybridization using the Human NGS WTA RNA (v1.0) probe set (NanoString Technologies). Slides were concurrently incubated with immunofluorescent antibodies, including pan-cytokeratin (AE-1/AE-3, Novus Biologicals, NBP2-33200AF532) to identify tumor cells and CD45 (2B11 + PD7/26, Novus Biologicals, NBP2-34528AF594) to detect immune cells. Next, slides were stained with the GeoMx Nuclear Stain (Syto13, Thermo Fisher Scientific, S7575), after which slides were loaded into the GeoMx Digital Spatial Profiler instrument (NanoString Technologies) and scanned. For each sample, two to five ROIs were selected at the tumor-stroma interface to represent the overall tumor microenvironment. ROI selection was reviewed and confirmed by a bone tumor pathologist (JVMGB). ROI areas were variable (0.145-0.481 mm^2^), with the aim of capturing at least 200 cells per segment to ensure representative gene expression data. A total of 63 ROIs were UV-illuminated twice to capture the pan-cytokeratin-positive tumor segment separately from all other cells, which were classified as stroma. CD45 staining was used to visualize immune cells and guide ROI selection but was not used for segment capture.

#### T cell infiltration from imaging mass cytometry data

We previously analyzed samples from the current cohort by means of imaging mass cytometry.^8^ T cell counts from that analysis were extracted to validate the GeoMx classification of the samples into inflamed (*n* = 7) and non-inflamed (*n* = 8) categories. One sample (L5436, classified as non-inflamed) was excluded due to the imaging mass cytometry data including both conventional and dedifferentiated tumor components, while the frozen sample analyzed by GeoMx comprised only of conventional chordoma.

#### MALDI-TOF-MSI

Concurrently with the preparation of the GeoMx slides, consecutive 5 μm tissue sections were cut from the frozen tissue samples, thaw-mounted onto conductive indium-tin-oxide (ITO)-coated glass slides (VisionTek Systems) and stored at -80°C until use. Before use, slides were placed in a vacuum freeze-drier for a minimum of 15 minutes. In total, samples from 13 of the 15 patients analyzed by GeoMx were available, and three recurrent tumor samples from two patients were additionally included to evaluate the preservation of lipid profiles over the course of disease.

Spatial metabolomics data was obtained using matrix-assisted laser desorption/ionization (MALDI)-MSI, using *N*-(1-naphthyl) ethylenediamine dihydrochloride (NEDC) (Sigma-Aldrich) as the matrix. A solution of 7 mg/mL NEDC in 70% MeOH (Actu-All Chemicals), 20% acetonitrile (Actu-All Chemicals) and 5% deionized water (MQ) was prepared. The tissue sections were coated with the matrix solution using a HTX M3+ sprayer (HTX Technologies). Detailed information about the matrix deposition step is available in Table S2.

Spatial metabolomics data was acquired at a spatial resolution of 20 μm using a rapifleX MALDI-TOF/TOF system (Bruker Daltonics) operating in reflectron mode, negative polarity, and set to detect a mass range of 60 to 1200 *m/z*. Each pixel was acquired with 50 shots at a laser repetition rate of 10 kHz.

#### High-resolution accurate-mass profiling acquisition

After spatial metabolomics data acquisition using the rapifleX MALDI-TOF/TOF, each tissue section was also subjected to high-resolution, accurate-mass (HRAM) profiling using a 15T solariX MALDI-FTICR mass spectrometer (Bruker Daltonics). The MALDI-FTICR was operated in negative-ion mode, using a 2M transient length, and set to detect a mass range of 400 to 1000 *m/z*. Each HRAM lipid/metabolite profile was collected from 25 laser shots, using a laser repetition rate of 120 Hz and with the laser focus set to ‘minimum’. After visual inspection of the spatial metabolomics results, HRAM profiles were acquired from regions that exhibited distinct lipid and metabolite signatures. After completing the spatial metabolomics and HRAM profiling, residual MALDI matrix was removed from the slides by ethanol washing and rehydration, followed by hematoxylin and eosin (H&E) staining according to standard diagnostic procedures. Finally, high resolution optical images of the H&E-stained tissue sections were recorded using a ZEISS Axio Scan.Z1 slide scanner (ZEISS).

#### PAS staining

Formalin-fixed paraffin-embedded (FFPE) tissue sections from corresponding tumors (*n* = 14) were stained using PAS and dPAS. PAS staining was performed using the Artisan PAS Stain Kit (AR165, Artisan, Agilent), while dPAS staining was carried out using the same kit in combination with alpha-amylase digestion using the Artisan Alpha-Amylase reagent (AR171, Artisan, Agilent). All staining procedures were executed on the Artisan Link Pro Special Staining System (Dako, Agilent) according to the manufacturer’s protocols. Comparison of PAS and dPAS staining demonstrated that the majority of the PAS signal in tumor cells was attributable to glycogen, as diastase digestion abolished most of the staining (Figure S8). Samples were subsequently classified as high (*n* = 9) or low (*n* = 5) based on the relative PAS staining intensity within the tumor cell compartment, as assessed by one observer (ZBE). The resulting groups were visualized in cumulative bar plots per identified GeoMx cluster using ggplot2 (R, v.3.5.1).

#### Immunohistochemistry

Immunohistochemical detection for Ki-67 (MIB-1, FLEX RTU, 1:1, Dako, GA626) was performed as described previously.^8^ FFPE tissue blocks corresponding to the same tumors used for the GeoMx/MALDI analyses (*n* = 14) were assessed. Given its overall low expression, Ki-67^+^ histological tumor regions were selected in QuPath (v.0.3.1) to quantify the percentage of Ki-67^+^ tumor cells per 1000 tumor cells.

#### IFN-γ treatment of chordoma cell lines

All chordoma cell lines (*n* = 4) were seeded in 50 μg/mL Collagen Type I (50202, Ibidi) coated 6-well plates at a cell density of 50.000 cells/well. After 11 days, cells were either not treated (mock) or treated with 100 U/mL IFN-γ (11343536, Immunotools). After one or three days of treatment, cells were harvested and pellets were stored at -80°C until RNA isolation was performed.

#### qPCR

Total RNA was isolated from the cell pellets using the NucleoSpin RNA Mini kit (Macherey-Nagel), and cDNA synthesis was performed using the Transcriptor First Strand cDNA Synthesis Kit (Roche). QPCR was performed to quantify four genes: *TBP* and *HPRT1* as housekeeping genes,^15^^,^^16^ *B2M* as positive control and *TBXT* as gene of interest. Transcript structure data from Ensembl indicates that *TBXT* (ENSG00000164458) encodes three protein-coding isoforms: *TBXT-201* (ENST00000296946) and *TBXT-203* (ENST00000366876) both include exon 6, while *TBXT-202* (ENST00000366871) excludes exon 6.^35^ Isoform-specific primers were therefore designed to distinguish between the “long” isoforms (*TBXT-201/203*) and the “short” isoform (*TBXT-202*), as well as primers targeting a region common to all *TBXT* transcripts (*TBXT* (all transcripts)). Primer sequences are provided in Table 1. The qPCR reactions were performed using iQ SYBR Green Supermix (Bio-Rad) and run on a C1000 Touch Thermal Cycler equipped with the CFX384 Touch Real-Time PCR Detection System (Bio-Rad). A standard PCR protocol was followed by annealing/extension at 59.2 °C for 45 seconds. All reactions were carried out in triplicate. Reactions were performed in 384-well Hard-Shell thin-wall PCR plates (Bio-Rad, HSP3805) sealed with Microseal 'B' adhesive film (Bio-Rad, MSB1001). Data were analyzed using CFX Maestro software (v.5.3.022.1030, Bio-Rad).

**Table 1:** Primer sequences

Gene/Transcript	Amplicon length (bp)	(5'-3')	
		Fwseq	Revseq
*B2M*	69	CTCCGTGGCCTTAGCTGTG	TTTGGAGTACGCTGGATAGCCT
*HPRT1*	94	TGACACTGGCAAAACAATGCA	GGTCCTTTTCACCAGCAAGCT
*TBP*	117	GAGCTGTGATGTGAAGTTTCC	TCTGGGTTTGATCATTCTGTAG
*TBXT-202*	94	CCTTGATGCAAAGGAAAGAAGTGA	GTTGTCAGAATAGGATTGGGAGT
*TBXT-201/203*	107	CCTTGATGCAAAGGAAAGAAGTGA	TGGTTCCAGGAAGAAGCCAC
*TBXT* (all transcripts)	93	CTCACCAACAAGCTCAACGG	TGGACCCCCAACTCTCACT

Fwseq = forward primer sequence; Revseq = reverse primer sequence; bps = base pairs.

### Quantification and statistical analysis

#### GeoMx data processing

All data processing and downstream analyses were performed in R (v.4.4.0) using the GeomxTools package (v.3.8.0). Quality control of the segments and the WTA probes was performed following NanoString’s analysis guideline. The used parameters and code are available on our lab’s GitHub at https://github.com/deMirandaLab/spatial-profiling-of-chordomas. In short, no segments were excluded after quality control, but 21 probes were excluded after performing the Grubb’s test. A limit of quantification was calculated for the detected genes in each segment, after which genes were excluded that were detected in less than 5% (*n* = 6) of all segments. This resulted in a final set of 8401 genes in 126 segments, with a minimum of 100 captured nuclei. The data was further processed with a Q3 quantile normalization and log_2_ transformation.

#### GeoMx data analysis

Tumor segments were compared with stroma segments through a differential gene expression analysis. A linear mixed model (LMM) was used to correct for varying number of segments per sample, as described by NanoString’s analysis guideline. The results were visualized in a volcano plot with ggplot2 (R, v.3.5.1) and the top 20 DEGs ranked by log_2_ fold change were selected for visualization. All DEGs were visualized in a heatmap using ComplexHeatmap (R, v.2.20.0). ssGSEA was performed using GSVA (R, v.1.52.3), which utilized an in-house curated WikiPathways gene list. The top differentially enriched biological pathways were identified comparing tumor and stroma segments through a similar LMM.

Next, segments were analyzed within their respective compartments. Both tumor and stroma segments were clustered into two groups through graph-based clustering (Louvain algorithm) using bluster (R, v.1.14.0), which were then visualized using UMAP. Differential gene expression analysis was again performed using an LMM, now comparing the identified clusters within their respective compartments. Again, the top 20 DEGs per cluster ranked by log_2_ fold change were selected for visualization in a volcano plot. All DEGs were visualized per compartment in heatmaps.

ssGSEA was subsequently performed for each compartment to compare pathway enrichment between the identified clusters. Differentially enriched pathways were identified using an LMM applied to ssGSEA scores, and the top 10% of pathways ranked by normalized enrichment score were visualized in heatmaps to guide biological interpretation. For all differential analyses, *p-*values were adjusted for multiple testing using the Benjamini–Hochberg method.

#### MALDI-TOF-MSI data processing

All spatial metabolomics data were imported into SCiLS Lab (v.2023b 11.01.14623, Bruker Daltonics) and subjected to baseline correction using the TopHat algorithm (width 200). The average spectrum of all tissues was exported from SCiLS Lab and used for peak picking in mMass (v.5.5.0) with a Signal-to-Noise Ratio (SNR) > 9, followed by removal of matrix derived peaks by image correlation to the principal matrix peak ([NEDC+Cl]^-^). Bisecting *k*-means clustering was employed in SCiLS Lab to differentiate on-tissue pixels from off-tissue pixels and areas without signal. Total Ion Count (TIC) normalized peak intensities and masses were exported as .csv files and processed further. Masses were internally calibrated to a list of abundant lipids and molecules commonly detected as negative ions when using NEDC as the matrix (Table S3). Spectra were normalized to the total ion count before further analysis. For small metabolite assignment, MALDI-TOF-MSI peaks were matched to the HMDB database (accessed 10/07/2024) with a mass error tolerance of ± 30 ppm allowing for [M-H]^-^ and [M+Cl]^-^ adducts.

#### Lipid/metabolite assignment based on high-resolution accurate-mass profiling

The HRAM profiles obtained from the MALDI-FTICR mass spectrometer were imported and processed in R (v.4.1) with MALDIquantForeign (v.0.13) and MALDIquant (v.1.20). Spectra were aligned, peak picked, and internally calibrated similarly to the MSI data (Table S4).

Resulting peaks were matched to the LIPIDMAPS LMSD database (accessed 14/01/2021) for phosphatidic acid (PA), phosphatidylethanolamine (PE), phosphatidylinositol (PI), phosphatidylserine (PS), and phosphatidylglycerol (PG), using a mass tolerance of ± 3 ppm and allowing for [M-H]^-^ and [M+Cl]^-^ adducts. This list was transferred to the lower mass resolution spatial metabolomics MALDI-TOF-MSI data, matching every MALDI-TOF-MSI peak with its analogous peak in the HRAM MALDI-FTICR spectra, using a tolerance of ± 40 ppm. Finally, all lipid assignments were manually curated to remove false positives and to ensure that all mass spectral peaks, which were used for the subsequent data analysis steps, corresponded to a single lipid species in the HRAM lipid profiles.

#### MALDI-TOF-MSI data analysis

Downstream analysis of the curated spatial metabolomics data was performed using Seurat (R, v.4.2). Per sample, data points (pixels) corresponding to holes in the tissue or necrotic tissue were removed after *k*-means clustering and subsequent clustering with Seurat. The lipid/metabolite abundance data of all samples was transformed into a single Seurat object followed by log normalization, after which a total of 1,223,810 pixels were clustered (Louvain algorithm) and visualized by a dimensionality reduction using UMAP. For differential analyses, clusters were classified as tumor or stroma by comparing their spatial localization with corresponding histopathological images, after which the mean lipid/metabolite intensity per region was calculated per sample.

#### qPCR data analysis

Average Ct values were calculated from technical triplicates. For each condition, ΔCt values were determined by subtracting the Ct of the housekeeping gene from that of the gene or transcript of interest. ΔΔCt values were then calculated by subtracting the ΔCt of the control condition (mock) from the ΔCt of the condition of interest. Fold changes in expression were computed using the 2^−ΔΔCt^ method. These calculations were performed separately using *HPRT1* and *TBP*, after which the average and standard deviation were calculated across both housekeeping genes. Boxplots were generated using ggplot2 (R, v.3.5.1) to visualize the normalized fold change in expression of the target genes and transcripts.

#### Statistical analysis

Unpaired Student’s t-tests were performed to compare features between the samples of the inflamed and non-inflamed clusters. The evaluated features included number of captured nuclei per segment (GeoMx), number of detected genes per segment (GeoMx), mean T cell density per mm^2^ (imaging mass cytometry), and the percentage of Ki-67^+^ tumor cells per 1000 tumor cells (immunohistochemistry). In the representative figures, statistical significance is indicated by asterisks as follows: ∗*p* < 0.05; ∗∗*p* < 0.01; ∗∗∗*p* < 0.001; ns = not significant.
