# Supplementary material for: Immune infiltration is associated with distinct transcriptional states and metabolic profiles in chordomas
Source: iScience. 2026 Apr 9;29(5):115668. doi: 10.1016/j.isci.2026.115668 (PMC13137147; doi:10.1016/j.isci.2026.115668)
Supplement: Document S1. Figures S1–S8 and Tables S1–S4 [file mmc1.pdf]

iScience, Volume *29*

Supplemental information

Immune infiltration is associated with distinct transcriptional states and metabolic profiles in chordomas

Siddh van Oost, Rick Ursem, Zeynep B. Erdem, Sara Cardoso, Sanne Venneker, Ruud van der Breggen, Inge H. Briaire-de Bruijn, Alwine B. Kruisselbrink, Wilco C. Peul, Karoly Szuhai, Robert J.P. van der Wal, Rene van Zeijl, Bram Heijs, Noel F.C.C. de Miranda, and Judith V.M.G. Bovee

Supplemental information index

Figure S1. GeoMx digital spatial profiling ROI selection

Figure S2. Differentially expressed genes in tumor and stroma segments

Figure S3. Differentially expressed genes in inflamed and non-inflamed stroma segments

Figure S4. Feature comparisons between inflamed and non-inflamed segments

Figure S5. Differentially expressed genes in inflamed and non-inflamed tumor segments

Figure S6. Differentially enriched pathways in inflamed and non-inflamed segments

Figure S7. Housekeeping genes remain stable after IFN-γ treatment

Figure S8. PAS/dPAS staining shows cytoplasmic glycogen

Table S1. Overview of the studied samples

Table S2. Matrix deposition details

Table S3. Molecular ions detected by FTICR used for calibration of the HRAM profiling data

Table S4. Molecular ions commonly detected by MALDI-MSI when using NEDC as matrix, used here for internal mass calibration

Supplemental reference list

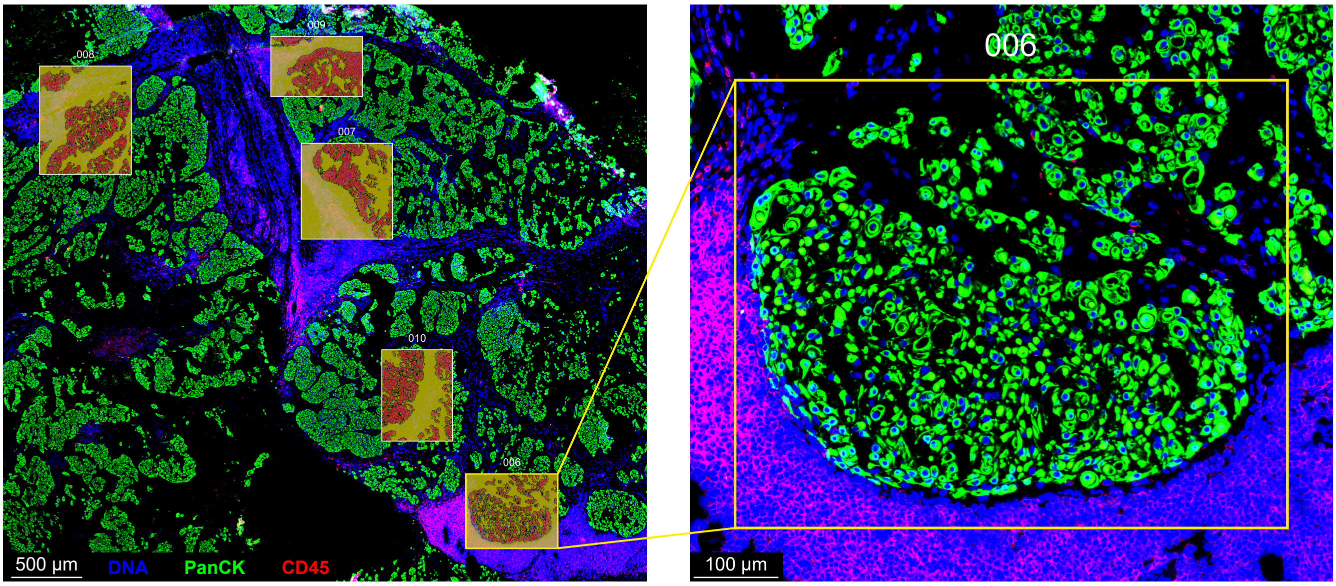

008
009
007
010
006
500 µm
DNA
PanCK
CD45
006
100 µm

Figure S1. GeoMx digital spatial profiling ROI selection. Representative immunofluorescence image of a chordoma, annotated with the selected regions of interest (ROIs) for the GeoMx experiment. The ROIs were segmented based on pan-cytokeratin (PanCK; green), thereby generating tumor and stroma segments. Stroma segments contained all other captured cells (DNA in blue), including immune cells (CD45 in red). Scale bars represent 500 µm in the left panel and 100 µm in the magnified panel on the right.

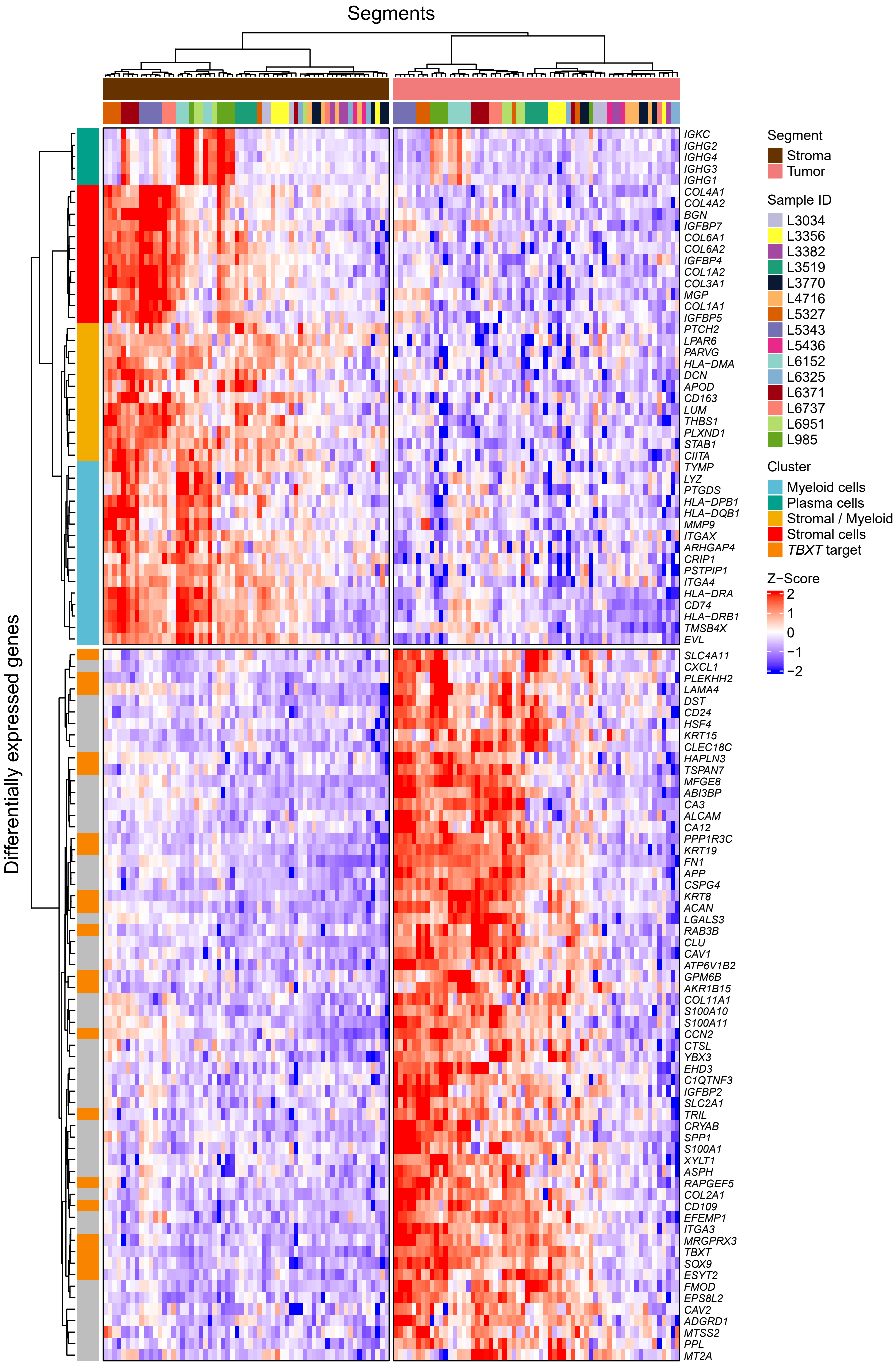
Segments
Differentially expressed genes
IGKC
IGHG2
IGHG4
IGHG3
IGHG1
COL4A1
COL4A2
BGN
IGFBP7
COL6A1
COL6A2
IGFBP4
COL1A2
COL3A1
MGP
COL1A1
IGFBP5
PTCH2
LPAR6
PARVG
HLA−DMA
DCN
APOD
CD163
LUM
THBS1
PLXND1
STAB1
CIITA
TYMP
LYZ
PTGDS
HLA−DPB1
HLA−DQB1
MMP9
ITGAX
ARHGAP4
CRIP1
PSTPIP1
ITGA4
HLA−DRA
CD74
HLA−DRB1
TMSB4X
EVL
SLC4A11
CXCL1
PLEKHH2
LAMA4
DST
CD24
HSF4
KRT15
CLEC18C
HAPLN3
TSPAN7
MFGE8
ABI3BP
CA3
ALCAM
CA12
PPP1R3C
KRT19
FN1
APP
CSPG4
KRT8
ACAN
LGALS3
RAB3B
CLU
CAV1
ATP6V1B2
GPM6B
AKR1B15
COL11A1
S100A10
S100A11
CCN2
CTSL
YBX3
EHD3
C1QTNF3
IGFBP2
SLC2A1
TRIL
CRYAB
SPP1
S100A1
XYLT1
ASPH
RAPGEF5
COL2A1
CD109
EFEMP1
ITGA3
MRGPRX3
TBXT
SOX9
ESYT2
FMOD
EPS8L2
CAV2
ADGRD1
MTSS2
PPL
MT2A
Segment
Stroma
Tumor
Sample ID
L3034
L3356
L3382
L3519
L3770
L4716
L5327
L5343
L5436
L6152
L6325
L6371
L6737
L6951
L985
Cluster
Myeloid cells
Plasma cells
Stromal / Myeloid
Stromal cells
TBXT target
Z−Score
2
1
0
−1
−2

Figure S2. Differentially expressed genes in tumor and stroma segments, related to Figure 1A. Heatmap displaying the differentially expressed genes (DEGs) comparing tumor (n = 63) with stroma (n = 63) segments. Segments are annotated for their tissue compartment and sample ID. Genes are grouped by their enriched compartment. In addition, stroma DEGs are annotated for cell type clusters and tumor DEGs are annotated if they are a target of *TBXT* [S1]. Differential gene expression analysis was performed as described in the **Figure 1** legend.

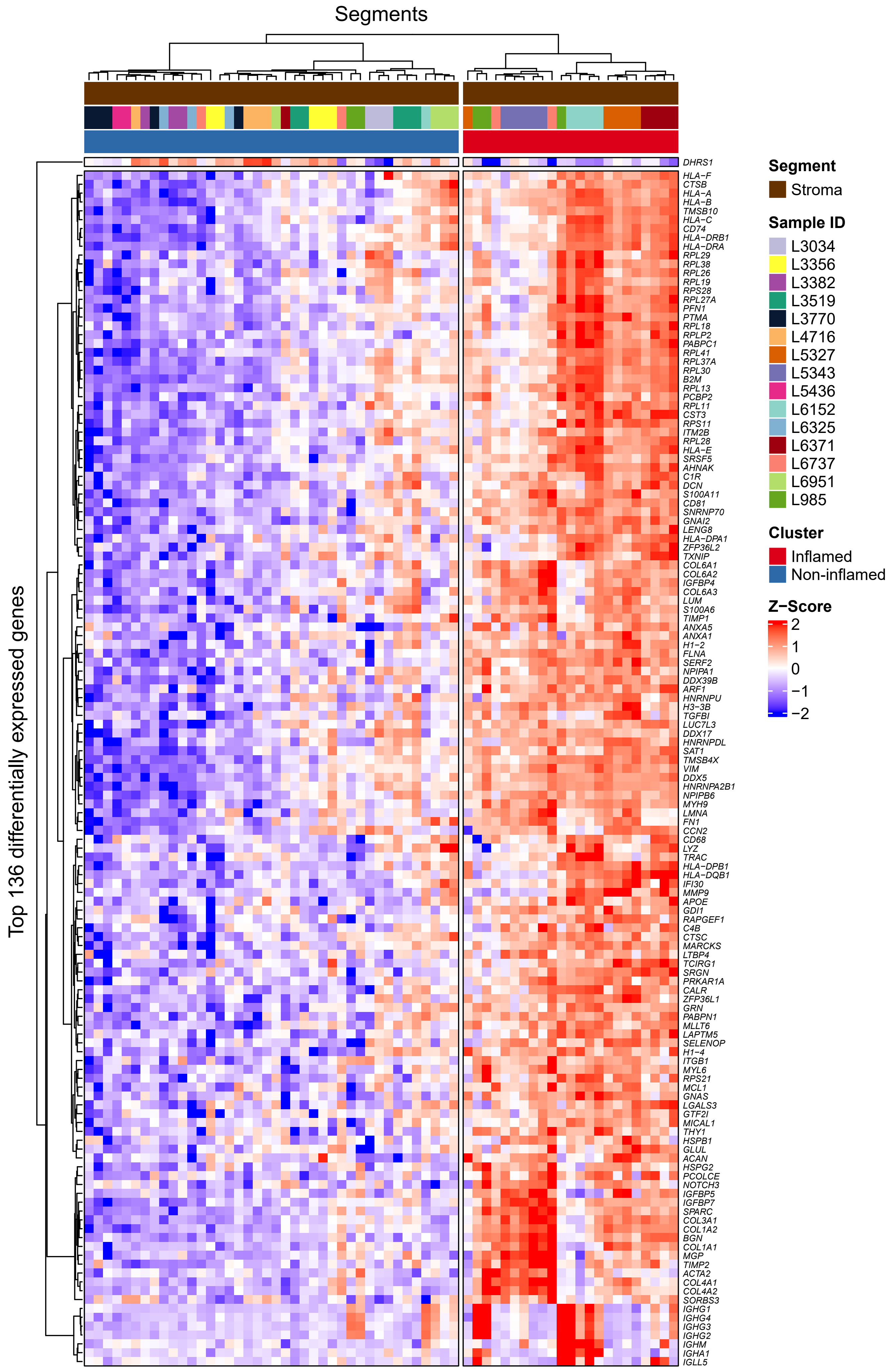

Segments
Top 136 differentially expressed genes
DHRS1
HLA-F
CTSB
HLA-A
HLA-B
TMSB10
HLA-C
CD74
HLA-DRB1
HLA-DRA
RPL29
RPL38
RPL26
RPL19
RPS28
RPL27A
PFN1
PTMA
RPL18
RPLP2
PABPC1
RPL41
RPL37A
RPL30
B2M
RPL13
PCBP2
RPL11
CST3
RPS11
ITM2B
RPL28
HLA-E
SRSF5
AHNAK
C1R
DCN
S100A11
CD81
SNRNP70
GNAI2
LENG8
HLA-DPA1
ZFP36L2
TXNIP
COL6A1
COL6A2
IGFBP4
COL6A3
LUM
S100A6
TIMP1
ANXA5
ANXA1
H1-2
FLNA
SERF2
NPIPA1
DDX39B
ARF1
HNRNPU
H3-3B
TGFBI
LUC7L3
DDX17
HNRNPDL
SAT1
TMSB4X
VIM
DDX5
HNRNPA2B1
NPIPB6
MYH9
LMNA
FN1
CCN2
CD68
LYZ
TRAC
HLA-DPB1
HLA-DQB1
IFI30
MMP9
APOE
GDI1
RAPGEF1
C4B
CTSC
MARCKS
LTBP4
TCIRG1
SRGN
PRKAR1A
CALR
ZFP36L1
GRN
PABPN1
MLLT6
LAPTM5
SELENOP
H1-4
ITGB1
MYL6
RPS21
MCL1
GNAS
LGALS3
GTF2I
MICAL1
THY1
HSPB1
GLUL
ACAN
HSPG2
PCOLCE
NOTCH3
IGFBP5
IGFBP7
SPARC
COL3A1
COL1A2
BGN
COL1A1
MGP
TIMP2
ACTA2
COL4A1
COL4A2
SORBS3
IGHG1
IGHG4
IGHG3
IGHG2
IGHM
IGHA1
IGLL5
Segment
Stroma
Sample ID
L3034
L3356
L3382
L3519
L3770
L4716
L5327
L5343
L5436
L6152
L6325
L6371
L6737
L6951
L985
Cluster
Inflamed
Non-inflamed
Z-Score
2
1
0
-1
-2

Figure S3. Differentially expressed genes in inflamed and non-inflamed stroma segments, related to Figure 1D. Heatmap displaying the DEGs comparing inflamed (n = 20) with non-inflamed (n = 43) stroma segments. Segments are annotated for their tissue compartment, sample ID and cluster. Genes are grouped by their enriched cluster. Differential gene expression analysis was performed as described in the **Figure 1** legend.

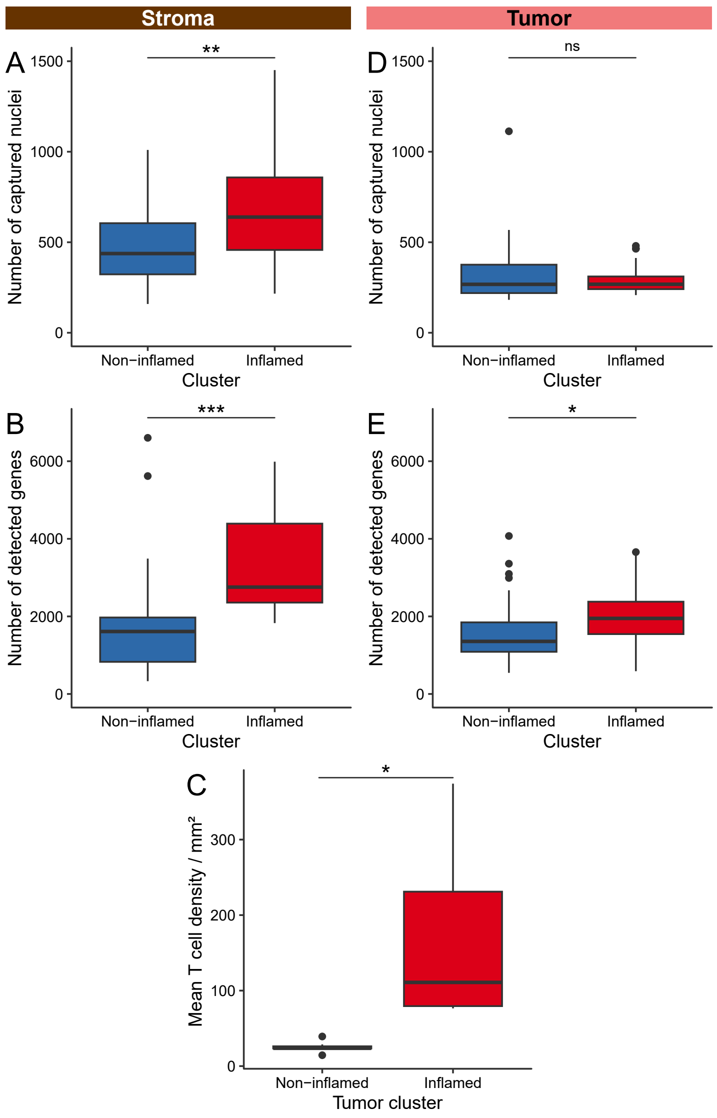

Stroma
Tumor
A
D
B
E
C
Number of captured nuclei
Number of detected genes
Mean T cell density / mm²
Non−inflamed
Inflamed
Cluster
Tumor cluster
**
ns
***
*
*

Figure S4. Feature comparisons between inflamed and non-inflamed segments, related to Figures 1C-1F. (A and B) Boxplots presenting the number of captured nuclei (A) and the number of detected genes (B) per stroma segment, grouped by assigned cluster (inflamed: $n = 20$; non-inflamed: $n = 43$). Statistical significance was assessed using unpaired Student's t-tests. *$p < 0.05$; **$p < 0.01$; ***$p < 0.001$; ns = not significant. (C) Boxplots presenting the mean T cell density per mm^2 per sample, as previously determined by imaging mass cytometry [S2], grouped by assigned tumor cluster (inflamed: $n = 7$; non-inflamed: $n = 7$). Statistical significance was assessed using an unpaired Student's t-test. (D and E) Boxplots presenting the number of captured nuclei (D) and the number of detected genes (E) per tumor segment, grouped by assigned cluster (inflamed: $n = 29$; non-inflamed: $n = 34$). Statistical significance was assessed using unpaired Student's t-tests.

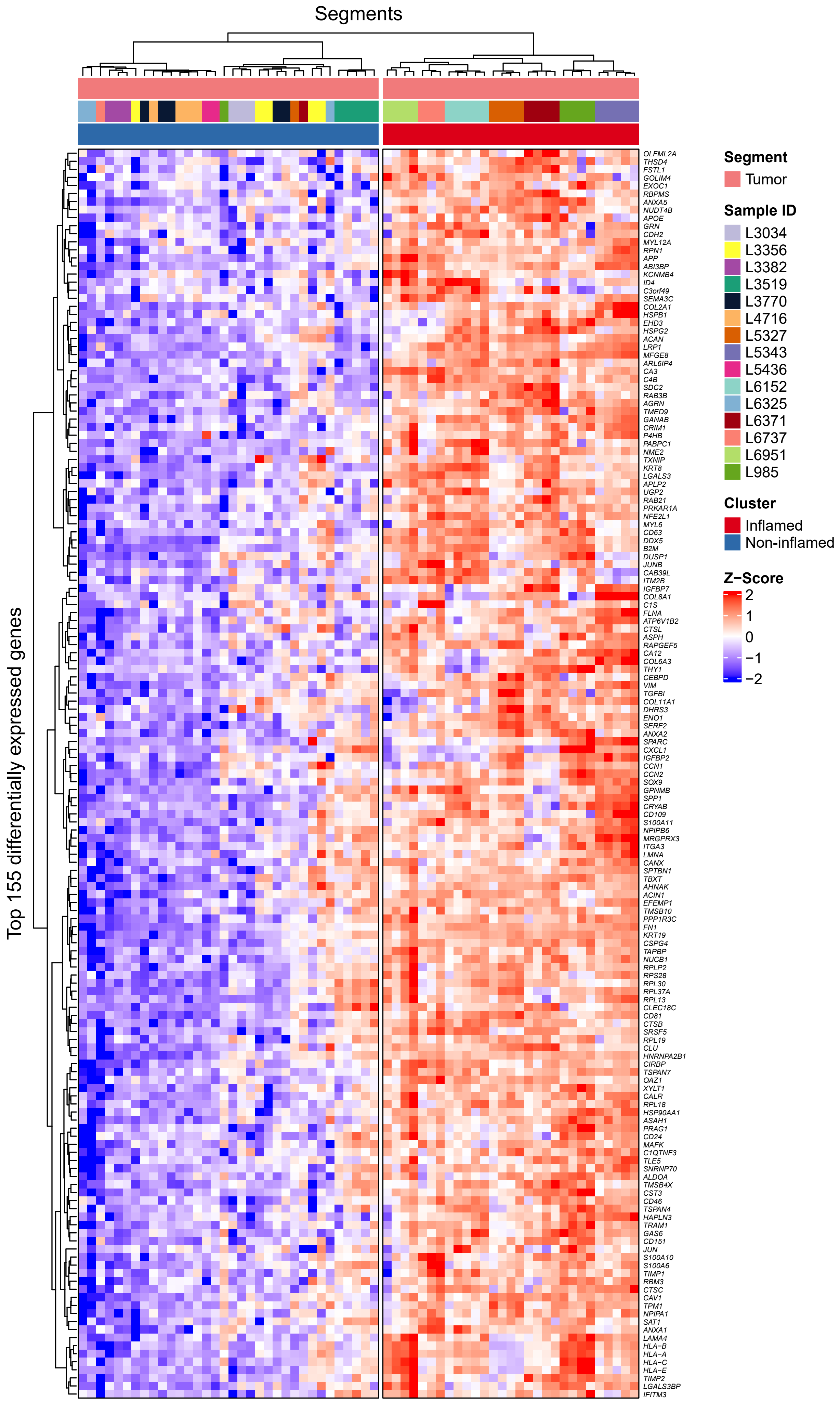
Segments
Top 155 differentially expressed genes
OLFML2A
THSD4
FSTL1
GOLIM4
EXOC1
RBPMS
ANXA5
NUDT4B
APOE
GRN
CDH2
MYL12A
RPN1
APP
ABI3BP
KCNMB4
ID4
C3orf49
SEMA3C
COL2A1
HSPB1
EHD3
HSPG2
ACAN
LRP1
MFGE8
ARL6IP4
CA3
C4B
SDC2
RAB3B
AGRN
TMED9
GANAB
CRIM1
P4HB
PABPC1
NME2
TXNIP
KRT8
LGALS3
APLP2
UGP2
RAB21
PRKAR1A
NFE2L1
MYL6
CD63
DDX5
B2M
DUSP1
JUNB
CAB39L
ITM2B
IGFBP7
COL8A1
C1S
FLNA
ATP6V1B2
CTSL
ASPH
RAPGEF5
CA12
COL6A3
THY1
CEBPD
VIM
TGFBI
COL11A1
DHRS3
ENO1
SERF2
ANXA2
SPARC
CXCL1
IGFBP2
CCN1
CCN2
SOX9
GPNMB
SPP1
CRYAB
CD109
S100A11
NPIPB6
MRGPRX3
ITGA3
LMNA
CANX
SPTBN1
TBXT
AHNAK
ACIN1
EFEMP1
TMSB10
PPP1R3C
FN1
KRT19
CSPG4
TAPBP
NUCB1
RPLP2
RPS28
RPL30
RPL37A
RPL13
CLEC18C
CD81
CTSB
SRSF5
RPL19
CLU
HNRNPA2B1
CIRBP
TSPAN7
OAZ1
XYLT1
CALR
RPL18
HSP90AA1
ASAH1
PRAG1
CD24
MAFK
C1QTNF3
TLE5
SNRNP70
ALDOA
TMSB4X
CST3
CD46
TSPAN4
HAPLN3
TRAM1
GAS6
CD151
JUN
S100A10
S100A6
TIMP1
RBM3
CTSC
CAV1
TPM1
NPIPA1
SAT1
ANXA1
LAMA4
HLA−B
HLA−A
HLA−C
HLA−E
TIMP2
LGALS3BP
IFITM3
Segment
Tumor
Sample ID
L3034
L3356
L3382
L3519
L3770
L4716
L5327
L5343
L5436
L6152
L6325
L6371
L6737
L6951
L985
Cluster
Inflamed
Non-inflamed
Z−Score
2
1
0
−1
−2

Figure S5. Differentially expressed genes in inflamed and non-inflamed tumor segments, related to Figure 1F. Heatmap displaying the DEGs comparing inflamed (n = 29) with non-inflamed (n = 34) tumor segments. Segments are annotated for their tissue compartment, sample ID and cluster. Differential gene expression analysis was performed as described in the **Figure 1** legend.

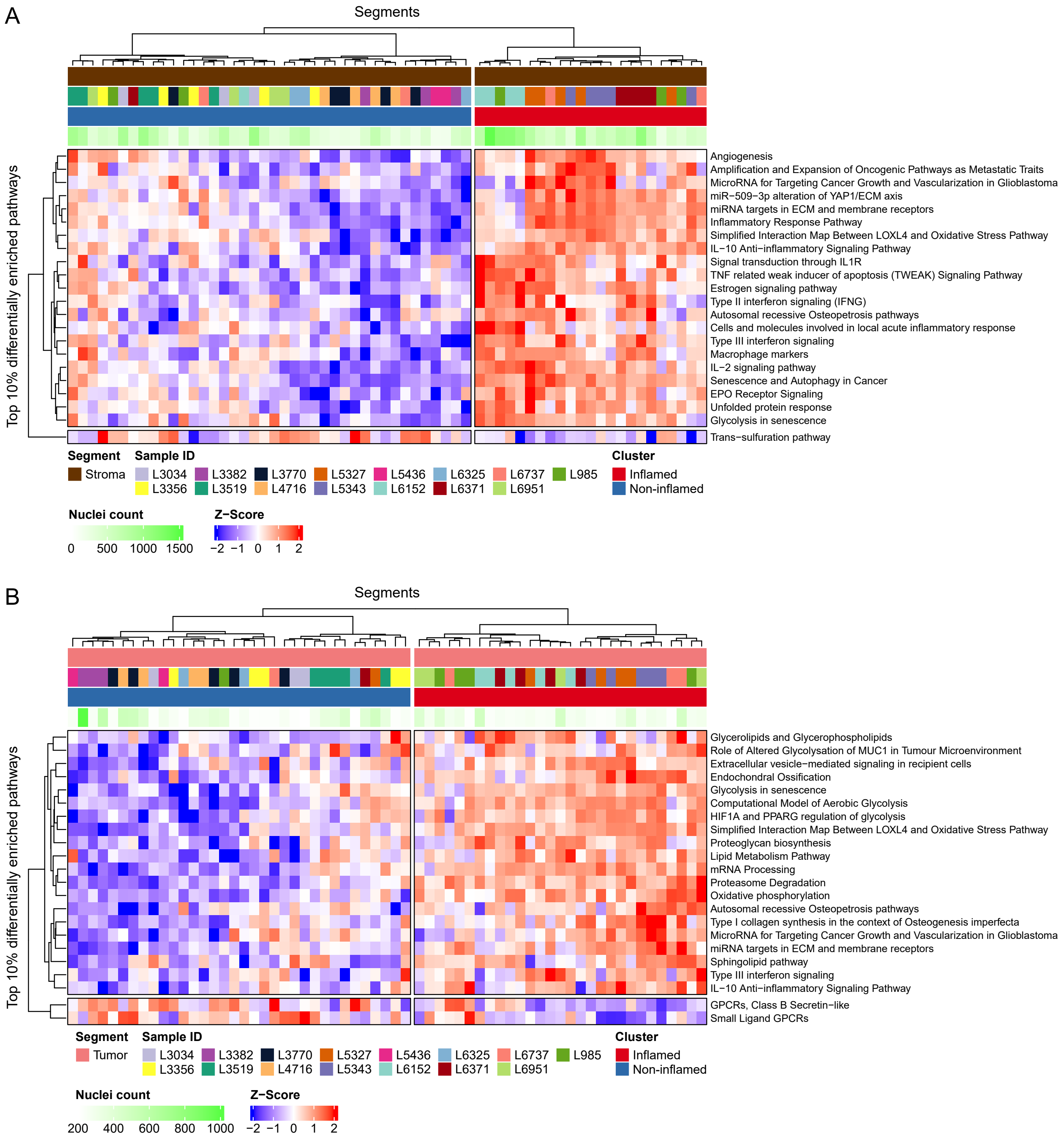
A
Segments
Top 10% differentially enriched pathways
Angiogenesis
Amplification and Expansion of Oncogenic Pathways as Metastatic Traits
MicroRNA for Targeting Cancer Growth and Vascularization in Glioblastoma
miR−509−3p alteration of YAP1/ECM axis
miRNA targets in ECM and membrane receptors
Inflammatory Response Pathway
Simplified Interaction Map Between LOXL4 and Oxidative Stress Pathway
IL−10 Anti−inflammatory Signaling Pathway
Signal transduction through IL1R
TNF related weak inducer of apoptosis (TWEAK) Signaling Pathway
Estrogen signaling pathway
Type II interferon signaling (IFNG)
Autosomal recessive Osteopetrosis pathways
Cells and molecules involved in local acute inflammatory response
Type III interferon signaling
Macrophage markers
IL−2 signaling pathway
Senescence and Autophagy in Cancer
EPO Receptor Signaling
Unfolded protein response
Glycolysis in senescence
Trans−sulfuration pathway
Segment
Stroma
Sample ID
L3034 L3382 L3770 L5327 L5436 L6325 L6737 L985
L3356 L3519 L4716 L5343 L6152 L6371 L6951
Cluster
Inflamed
Non-inflamed
Nuclei count
0 500 1000 1500
Z−Score
−2 −1 0 1 2
B
Segments
Top 10% differentially enriched pathways
Glycerolipids and Glycerophospholipids
Role of Altered Glycolysation of MUC1 in Tumour Microenvironment
Extracellular vesicle−mediated signaling in recipient cells
Endochondral Ossification
Glycolysis in senescence
Computational Model of Aerobic Glycolysis
HIF1A and PPARG regulation of glycolysis
Simplified Interaction Map Between LOXL4 and Oxidative Stress Pathway
Proteoglycan biosynthesis
Lipid Metabolism Pathway
mRNA Processing
Proteasome Degradation
Oxidative phosphorylation
Autosomal recessive Osteopetrosis pathways
Type I collagen synthesis in the context of Osteogenesis imperfecta
MicroRNA for Targeting Cancer Growth and Vascularization in Glioblastoma
miRNA targets in ECM and membrane receptors
Sphingolipid pathway
Type III interferon signaling
IL−10 Anti−inflammatory Signaling Pathway
GPCRs, Class B Secretin−like
Small Ligand GPCRs
Segment
Tumor
Sample ID
L3034 L3382 L3770 L5327 L5436 L6325 L6737 L985
L3356 L3519 L4716 L5343 L6152 L6371 L6951
Cluster
Inflamed
Non-inflamed
Nuclei count
200 400 600 800 1000
Z−Score
−2 −1 0 1 2

Figure S6. Differentially enriched pathways in inflamed and non-inflamed segments, related to Figures 1C-1F. (A and B) Heatmaps displaying the top differentially enriched pathways in inflamed (n = 20) versus non-inflamed (n = 43) stroma segments (A) and inflamed (n = 29) versus non-inflamed (n = 34) tumor segments (B). Pathways are grouped by their enriched cluster. In addition, segments are annotated for their tissue compartment, sample ID, cluster, and nuclei count. Statistical significance was determined using a linear mixed model correcting for sample ID and the top 10% of pathways ranked by normalized enrichment score were selected for visualization.

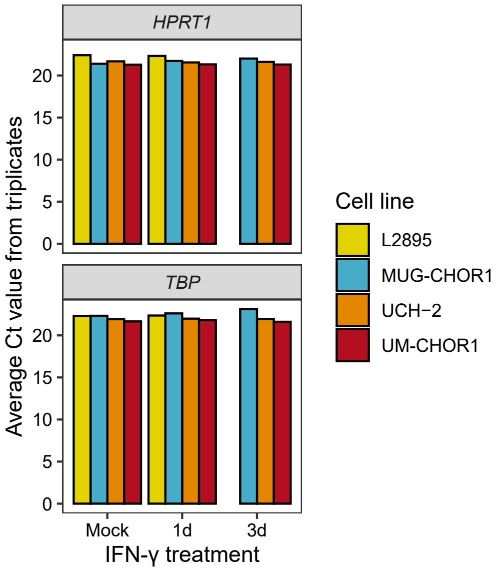

HPRT1
TBP
Average Ct value from triplicates
20
15
10
5
0
Mock
1d
3d
IFN-γ treatment
Cell line
L2895
MUG-CHOR1
UCH−2
UM-CHOR1

Figure S7. Housekeeping genes remain stable after IFN-γ treatment, related to Figure 4. Average Ct values for *HPRT1* and *TBP* from technical triplicates for all chordoma cell lines (*n* = 4) and treatment condition. Abbreviations: 1d = 1 day; 3d = 3 days.

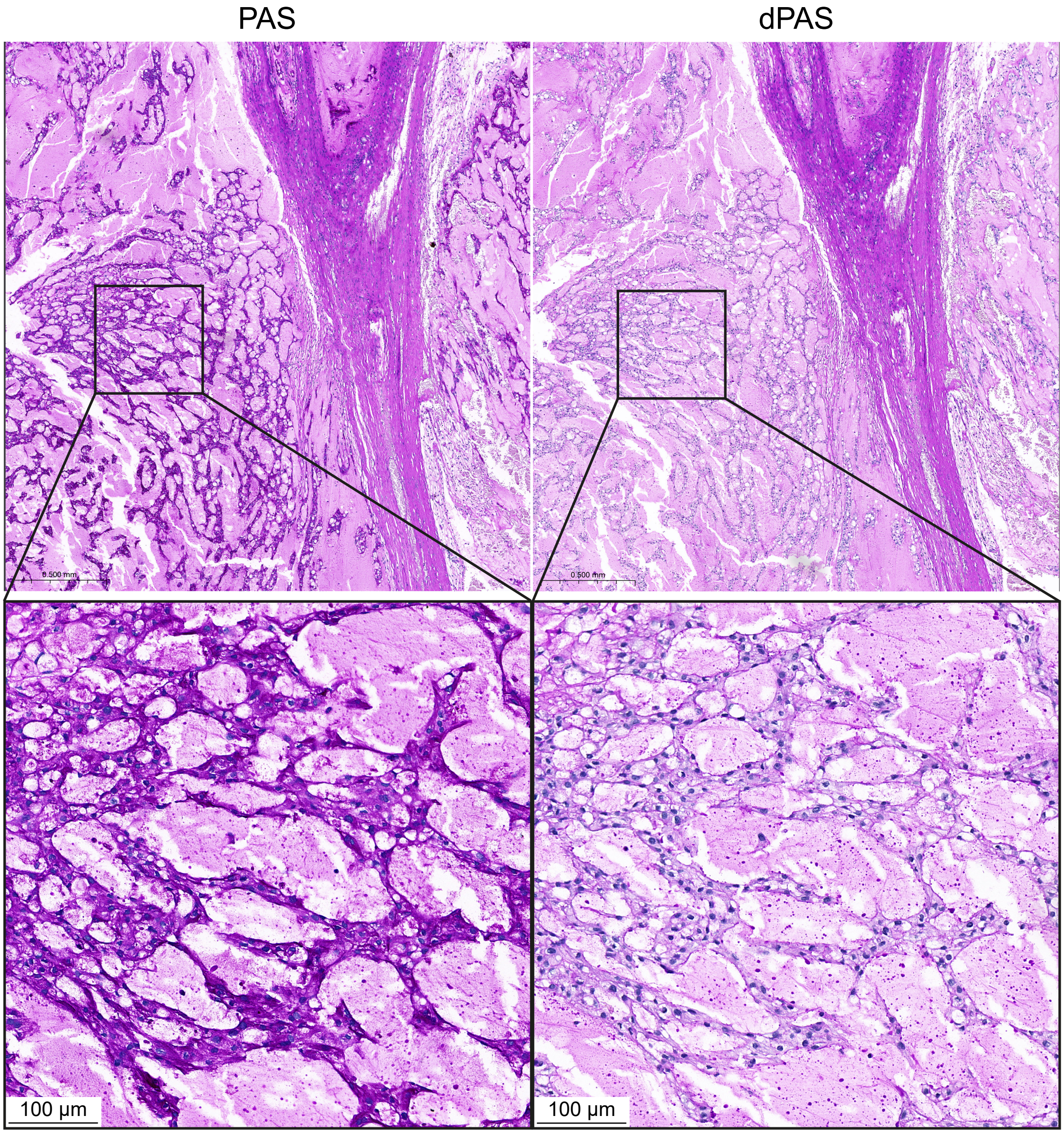
PAS
dPAS
100 µm
100 µm

Figure S8. PAS/dPAS staining shows cytoplasmic glycogen, related to STAR Methods. Periodic acid-Schiff (PAS) and diastase-PAS (dPAS) staining from corresponding areas in the same sample on consecutive formalin-fixed paraffin-embedded slides, showing the loss of glycogen after digestion with diastase. Scale bars represent 0.5 mm in the top panels and 100 μm in the magnified panels at the bottom.

Tables S1-S4, related to STAR Methods

Table S1. Overview of the studied samples. RT = radiotherapy, ST = soft tissue.

Sample ID	Patient ID	Anatomical location	Sample type	Neoadjuvant RT	GeoMx	MALDI
L985	P1	Sacrum	Recurrence		Yes	Yes
L2038	P1		Recurrence			Yes
L4336	P1		Recurrence			Yes
L3034		Sacrum	Primary		Yes	Yes
L3356		Sacrum	Primary		Yes	
L3382		Clivus	Primary		Yes	Yes
L3519		Sacrum	Primary		Yes	Yes
L3770		Sacrum	Primary		Yes	Yes
L4716		Sacrum	Primary		Yes	Yes
L5327		Sacrum	Primary	Yes	Yes	Yes
L5343	P2	Sacrum	Primary		Yes	Yes
L6448	P2		Recurrence			Yes
L5436		Mobile Spine (Lumbar)	Primary		Yes	Yes
L6152		Mobile Spine (Left Flank ST)	Recurrence		Yes	Yes
L6325		Sacrum	Primary	Yes	Yes	Yes
L6371		Sacrum	Primary		Yes	Yes
L6737		Clivus	Primary		Yes	Yes
L6951		Sacrum (Coccyx)	Primary	Yes	Yes	

Table S2. Matrix deposition details.

Parameter	Setting
Nozzle temperature (C)	60
Passes	20
Flowrate (µL / min)	80
Velocity (mm / min)	2000
Track spacing (mm)	3
Pattern	CC
Nitrogen pressure (psi)	10
Drying time between passes (s)	30

Table S3. Molecular ions detected by FTICR used for calibration of the HRAM profiling data.

Compound	Accurate mass
LPE(18:1)	478.2939
LPE(22:6)	524.2783
GSSG	611.1452
Heme	615.1700
PE(36:2)	742.5392
PE(38:4)	766.5392
PS(38:4)	810.5290
PI(38:4)	885.5498

Table S4. Molecular ions commonly detected by MALDI-MSI when using NEDC as matrix, used here for internal mass calibration.

**Compound**	**Accurate mass**
Taurine	124.006841
Phosphoethanolamine	140.01187
L-Glutamic acid	146.045334
F6P	259.022446
Inosine	267.073494
Linoleic acid	279.232953
Oleic acid	281.248603
Stearic acid	283.264253
Arachidonic acid	303.232953
Glutathione	306.076532
AMP	346.055811
ADP	426.022144
LPE(18:1)	478.293915
LPE(22:6)	524.2783
GSSG	611.145272
Heme	615.170018
PE(36:2)	742.53923
PE(38:4)	766.53923
PS(38:4)	810.52906
PI(38:4)	885.549856

Supplemental reference list

S1. Nelson, A.C., et al., *An integrated functional genomics approach identifies the regulatory network directed by brachyury (T) in chordoma.* J Pathol, 2012. **228**(3): p. 274-85.

S2. van Oost, S., et al., *Multimodal profiling of chordoma immunity reveals distinct immune contextures.* J Immunother Cancer, 2024. **12**(1).
